# *In vitro* susceptibility of *Clostridium difficile* to SMT19969 and comparators, as well as the killing kinetics and post-antibiotic effects of SMT19969 and comparators against *C. difficile*

**DOI:** 10.1093/jac/dkv006

**Published:** 2015-02-03

**Authors:** D. Corbett, A. Wise, S. Birchall, P. Warn, S. D. Baines, G. Crowther, J. Freeman, C. H. Chilton, J. Vernon, M. H. Wilcox, R. J. Vickers

**Affiliations:** 1 Evotec (UK), Williams House, Manchester Science Park, Lloyd Street North, Manchester M15 6SE, UK; 2 Department of Life and Medical Sciences, University of Hertfordshire, Hatfield AL10 9AB, UK; 3 Department of Microbiology, Leeds Teaching Hospitals NHS Trust and Healthcare Associated Infections Research Group, The University of Leeds, Old Medical School, Leeds LS1 3EX, UK; 4 Summit plc, 85b Park Drive, Milton Park, Abingdon, Oxfordshire OX14 4RY, UK

**Keywords:** *C. difficile*, PAE, antimicrobial

## Abstract

**Objectives:**

SMT19969 is a novel antimicrobial under clinical development for the treatment of *Clostridium difficile* infection (CDI). The objective was to determine the comparative susceptibility of 82 *C. difficile* clinical isolates (which included ribotype 027 isolates and isolates with reduced metronidazole susceptibility) to SMT19969, fidaxomicin, vancomycin and metronidazole and to determine the killing kinetics and post-antibiotic effects of SMT19969, fidaxomicin and vancomycin against *C. difficile*.

**Methods:**

MICs were determined by agar incorporation. Killing kinetics and post-antibiotic effects were determined against *C. difficile* BI1, 630 and 5325 (ribotypes 027, 012 and 078, respectively).

**Results:**

SMT19969 showed potent inhibition of *C. difficile* (MIC_90_=0.125 mg/L) and was markedly more active than either metronidazole (MIC_90_ = 8 mg/L) or vancomycin (MIC_90_ = 2 mg/L). There were no differences in susceptibility to SMT19969 between different ribotypes. Fidaxomicin was typically one doubling dilution more active than SMT19969 and both agents maintained activity against isolates with reduced susceptibility to metronidazole. In addition, SMT19969 was bactericidal against the *C. difficile* strains tested, with reductions in viable counts to below the limit of detection by 24 h post-inoculation. Vancomycin was bacteriostatic against all three strains. Fidaxomicin was bactericidal although reduced killing was observed at concentrations <20 × MIC against *C. difficile* BI1 (ribotype 027) compared with other strains tested.

**Conclusions:**

These data demonstrate that SMT19969 is associated with potent and bactericidal activity against the strains tested and support further investigation of SMT19969 as potential therapy for CDI.

## Introduction

*Clostridium difficile* infection (CDI) is a significant cause of morbidity and mortality in both the acute care setting and the wider healthcare system.^1,2^ The global increase in the incidence of CDI is driven, in part, by the emergence of fluoroquinolone-resistant ribotype 027 *C. difficile* strains,^3^ which continue to account for ∼30% of CDI cases in North America.^4^ These strains are associated with poor outcomes, including reduced cure rates and increased rates of recurrent disease.^5,6^ Although the prevalence of ribotype 027 was thought to have declined in Europe,^7^ the recent EUCLID study demonstrates the dominance of ribotype 027 in Central and Eastern Europe.^8^ Also, new hyper-virulent strains such as ribotype 244 continue to emerge.^9^

CDI pathogenesis is associated with antimicrobial use that causes reduced diversity of the gut microbiota, thus reducing the host's ability to resist colonization by, and expansion of, *C. difficile*. These conditions allow *C. difficile* spores to germinate, with resultant toxin production leading to disease symptoms. Vancomycin and metronidazole, the mainstay antibiotics used in CDI, have been shown to cause further collateral damage to the gut microbiota,^10–12^ and analysis of the gut microbiome of CDI patients has shown that recurrent disease is associated with a markedly decreased diversity in the bacterial populations of the gut.^13^ As such, therapeutic approaches that minimize further deleterious effects to the gut microbiota may reduce rates of recurrent CDI.

SMT19969 is a novel antimicrobial under specific development for CDI that shows potent inhibition of *C. difficile*, but is associated with minimal growth inhibition of both Gram-positive and Gram-negative components of the indigenous gut microflora;^14,15^ such focused activity may result in reduced rates of recurrent disease. The following studies further assessed and compared growth inhibition by, and the *in vitro* pharmacodynamics of, SMT19969, fidaxomicin, vancomycin and metronidazole against *C. difficile*.

## Materials and methods

### Test agents

SMT19969 (supplied by Summit plc, Abingdon, UK), fidaxomicin [obtained from Cubist Pharmaceuticals, Lexington, MA, USA (for MIC determination) and from Santa Cruz Biotechnology, Santa Cruz, CA, USA (for kill curve and post-antibiotic effect, PAE, assays)] and metronidazole (supplied by Sigma) were reconstituted in DMSO prior to further dilution. Vancomycin (supplied by Sigma) stock solutions were prepared in water.

### Bacterial strains

Susceptibility testing was carried out using *C. difficile* clinical isolates collected in the UK from subjects with CDI. All isolates had been submitted to the *C. difficile* ribotyping network. A total of 82 *C. difficile* isolates were assessed across five panels comprising 30 distinct isolates of different PCR ribotypes (referred to as the genotypically distinct group), 10 PCR ribotype 001 isolates, 11 PCR ribotype 027 isolates, 10 PCR ribotype 106 isolates and 21 PCR ribotype 001 isolates showing reduced metronidazole susceptibility (MIC 4–8 mg/L).^16^ *Staphylococcus aureus* ATCC 29213, *Bacteroides fragilis* ATCC 25285 and *C. difficile* PCR ribotype 010 E4 were used as control organisms. *C. difficile* E4 is a non-toxigenic PCR ribotype 010 internal control strain.^17^

Kill curve and PAE studies were performed using *C. difficile* 630 (ATCC BAA 1382; ribotype 012), BI1 (NCTC 13366; ribotype 027) and 5325 (ATCC BAA-1875; ribotype 078).

### MICs

Comparative susceptibility testing was carried out on Wilkins Chalgren agar due to the superior growth compared with Brucella agar and also the ability to detect reduced susceptibility to metronidazole.^16^ MICs were determined by agar incorporation according to a previously validated method.^16^ All isolates were tested in duplicate for susceptibility to SMT19969, fidaxomicin, vancomycin and metronidazole.

For the kill curve and PAE studies, MIC values (Table 1) were established for SMT19969, fidaxomicin and vancomycin by broth microdilution using BHIS medium (brain heart infusion broth supplemented with 5 g/L yeast extract and 0.025% l-cysteine); this medium was used in order to be consistent with later experiments.

**Table 1.** Reference MICs established by broth microdilution for killing kinetics and PAE studies

*C. difficile* strain (ribotype)	MIC (mg/L)		
	SMT19969	vancomycin	fidaxomicin
BI1 (027)	0.125	4	0.25
630 (012)	0.125	4	0.25
5325 (078)	0.125	1	0.25

### Killing kinetics

Cultures of *C. difficile* were prepared by inoculating fresh, pre-reduced BHIS medium with a single colony of the required strain. Following overnight (18–20 h) incubation, cultures were back-diluted 1: 100 into fresh BHIS broth (∼10^6^ cfu/mL) containing either DMSO (1% v/v; vehicle) or 1, 2, 5, 10 or 20 × MIC of SMT19969, vancomycin or fidaxomicin. Viable counts were determined at 0, 2, 4, 6, 8 and 24 h post-inoculation on BHIS agar. Data presented are the means of triplicate experiments. The limit of detection (LOD) for these assays was 500 cfu/mL. A ≥3 log_10_ reduction in viability relative to the starting inoculum was considered bactericidal.

### PAE

Cultures of *C. difficile* were prepared in 15 mL polypropylene centrifuge tubes in the presence of DMSO or antibiotic, as described above. Cultures were incubated for 1 h before being collected by centrifugation (4696 **g**, 5 min, 25°C), washed once with BHIS to remove test articles and finally re-suspended in fresh BHIS. For assays involving exposure to fidaxomicin, cultures were transferred to sterile glass bijou tubes following removal of the test article in order to minimize drug carryover due to non-specific binding as previously reported.^18^ Centrifugation was the only step performed outside the anaerobic workstation. At this point, samples were removed for determination of viable counts (0 h) and further samples were removed for counting after 2, 4, 6, 8 and 24 h. The LOD for these experiments was 50 cfu/mL. The PAE of each test article was defined as PAE = *T* − *C*, where T is the time required for determination of the viable count to increase 10-fold over the post-washing viable count in the presence of antibiotic, and *C* is the time required for the viable count to increase 10-fold over the post-washing viable count in the absence of antibiotic. The mean values obtained from three independent experiments were used for *T* and *C*. PAE (in hours) was estimated to the nearest timepoint. Where the PAE endpoint was reached overnight, the endpoint was defined as the limits of the PAE (i.e. 8–20 h). No interpolation between timepoints was attempted.

## Results

### Susceptibility testing

The comparative susceptibilities of 82 *C. difficile* clinical isolates to SMT19969, fidaxomicin, metronidazole and vancomycin are shown in Table 2. Vancomycin generally maintained a consistent level of activity against the majority of the panels assessed with an MIC_90_ of 2 mg/L (typical range = 0.5–4 mg/L) for each panel except for the metronidazole-susceptible ribotype 001 panel where an MIC_90_ = 4 mg/L was recorded. Although the vast majority of the 82 isolates had vancomycin MIC values of 1 or 2 mg/L, seven isolates (ribotypes 056, 001, 027 and 106) showed an elevated MIC value of 4 mg/L. Susceptibility to metronidazole was more varied across the panels. For ribotypes 027 and 106, MIC_50_ values were one dilution higher than for the metronidazole-susceptible ribotype 001 panel, and two dilutions higher than MIC_50_ values recorded for the genotypically distinct group.

**Table 2.** MICs of SMT19969, fidaxomicin, metronidazole and vancomycin for 82 *C. difficile* clinical isolates

Panel (*n*)	Agent	MIC range (mg/L)	MIC_50_ (mg/L)	MIC_90_ (mg/L)
Genotypically distinct (30)^a^	SMT19969	0.06–0.125	0.125	0.125
	vancomycin	1–4	2	2
	metronidazole	0.25–2	0.5	2
	fidaxomicin	0.008–0.125	0.03	0.06
Ribotype 001 (10)	SMT19969	0.06–0.125	0.125	0.125
	vancomycin	0.5–4	1	4
	metronidazole	0.125–1	1	1
	fidaxomicin	0.008–0.06	0.03	0.06
Ribotype 027 (11)	SMT19969	0.125–0.25	0.125	0.125
	vancomycin	0.5–4	1	2
	metronidazole	1–2	2	2
	fidaxomicin	0.03–0.06	0.06	0.06
Ribotype 106 (10)	SMT19969	0.125–0.25	0.125	0.125
	vancomycin	0.5–4	1	2
	metronidazole	1–2	2	2
	fidaxomicin	0.03–0.125	0.06	0.125
Reduced metronidazole susceptibility (21)^b^	SMT19969	0.06–0.125	0.125	0.125
	vancomycin	0.5–4	1	2
	metronidazole	4–8	4	8
	fidaxomicin	0.015–0.03	0.03	0.03
Overall total (82)	SMT19969	0.06–0.25	0.125	0.125
	vancomycin	0.5–4	1	2
	metronidazole	0.125–8	2	8
	fidaxomicin	0.008–0.125	0.03	0.06

^a^Thirty distinct isolates of different PCR ribotype.

^b^Ribotype 001.

Both fidaxomicin and SMT19969 showed potent activity against the 82 isolates tested, with MIC_90_ values of 0.06 mg/L (range = 0.008–0.125 mg/L) and 0.125 mg/L (range = 0.06–0.25 mg/L), respectively, which was typically 16–32-fold lower than the MIC_90_ values recorded for either vancomycin against the 82 isolates or metronidazole against the metronidazole-susceptible isolates. Fidaxomicin showed increased potency compared with SMT19969 with MIC_90_ values one dilution and MIC_50_ values two dilutions lower than those recorded for SMT19969. SMT19969 activity was consistent across different ribotypes, including ribotype 027 isolates; the MIC_90_ for each panel of clinical isolates was 0.125 mg/L. Similarly, fidaxomicin showed highly consistent activity against the strains tested, although it was slightly less active against ribotype 106 compared with the other isolates. For the PCR ribotype 001 isolates showing reduced susceptibility to metronidazole, both vancomycin and SMT19969 maintained activity levels comparable to those observed against the metronidazole-susceptible ribotype 001 strains, whilst fidaxomicin MIC_90_ values were one dilution lower (0.03 versus 0.06 mg/L).

Overall SMT19969 showed potent growth inhibition of the 82 isolates tested, with an MIC_90_ of 0.125 mg/L, which was 16-fold lower than the MIC_90_ values recorded for vancomycin against the 82 isolates and the 61 metronidazole-susceptible isolates.

### Killing kinetics

**Figure 1.**
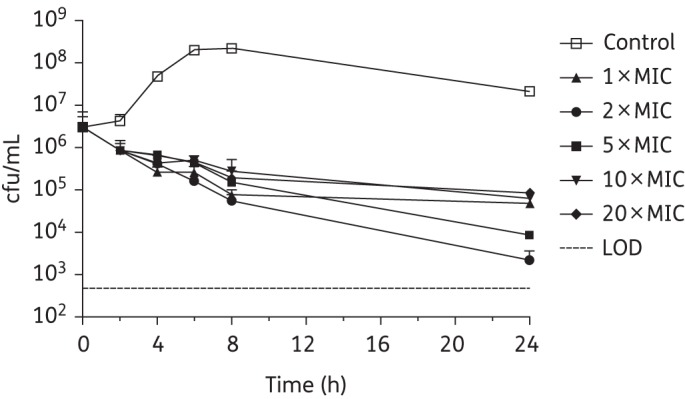
Twenty-four hour time–kill curves for vancomycin against *C. difficile* BI1. Data are the means (+SD) of triplicate experiments.

**Figure 2.**
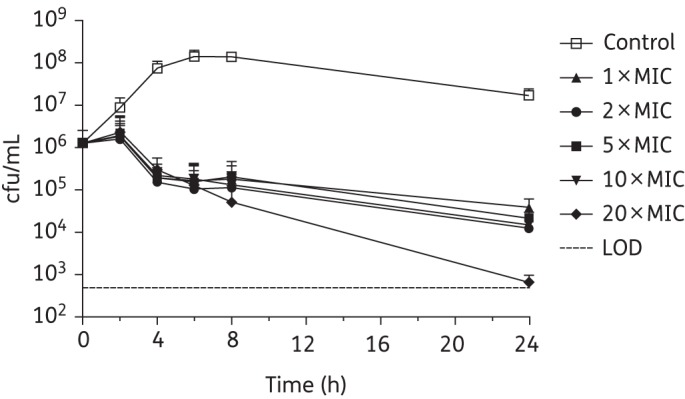
Twenty-four hour time–kill curves for fidaxomicin against *C. difficile* BI1. Data are the means (+SD) of triplicate experiments.

**Figure 3.**
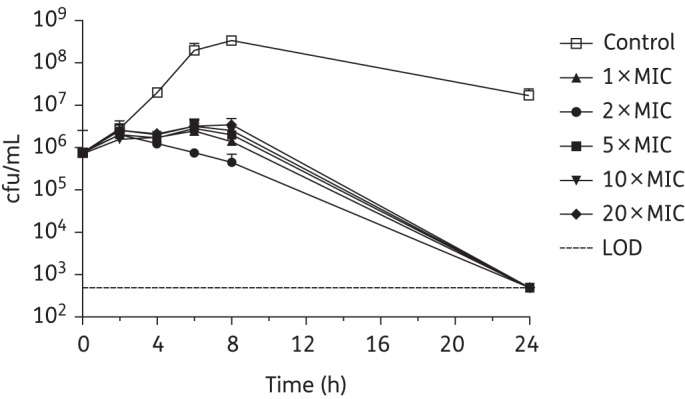
Twenty-four hour time–kill curves for SMT19969 against *C. difficile* BI1. Data are the means (+SD) of triplicate experiments.

The comparative killing kinetics of SMT19969, vancomycin and fidaxomicin were assessed against *C. difficile* strains BI1 (ribotype 027), 630 (ribotype 012) and 5325 (ribotype 078). Kill curves for vancomycin, fidaxomicin and SMT19969 against *C. difficile* BI1 are shown in Figures 1–3, with reductions in cfu/mL for *C. difficile* 630 and 5325 following 24 h of exposure to antibiotic shown in Table 3. It should be noted that the starting inoculum for experiments with fidaxomicin and *C. difficile* 630 and 5325 was somewhat lower (3.72 × 10^5^ and 2.17 × 10^5^ cfu/mL, respectively) than for vancomycin (1.94 × 10^6^ and 9.00×10^5^ cfu/mL) or SMT19969 (3.28 × 10^6^ and 1.42 × 10^6^ cfu/mL). Starting inocula for experiments with *C. difficile* BI1 for SMT19969, fidaxomicin and vancomycin were 7.50 × 10^5^, 1.30 × 10^6^ and 3.17 × 10^6^ cfu/mL, respectively.

**Table 3.** Mean log_10_ cfu/mL reductions for *C. difficile* 630 and 5325 following 24 h of exposure to SMT19969, vancomycin or fidaxomicin

Strain	Agent	Mean log_10_ reductions in cfu/mL				
		1 × MIC	2 × MIC	5 × MIC	10 × MIC	20 × MIC
630	SMT19969	3.61	2.17	>3.82^a^	>3.82^a^	>3.82^a^
	vancomycin	3.25	3.22	2.99	3.46	3.59
	fidaxomicin	2.45	>2.87^a^	>2.87^a^	>2.87^a^	>2.87^a^
5325	SMT19969	2.60	1.62	2.73	3.16	3.33
	vancomycin	1.14	2.65	1.82	1.81	1.46
	fidaxomicin	2.41	2.16	>2.64^a^	>2.64^a^	>2.64^a^

^a^Reduced to below LOD.

Vancomycin generally achieved a slow reduction in bacterial counts of *C. difficile* BI1, resulting in a 1.5–2.5 log_10_ reduction in viability after 24 h (Figure 1); a 3.1 log_10_ reduction in cfu/mL was observed at 24 h at 2 × MIC. Similarly, fidaxomicin at 1–10 × MIC was bacteriostatic against *C. difficile* BI1 with a 1.5–2.0 log_10_ reduction in viable counts. At 20 × MIC, fidaxomicin was bactericidal, resulting in a 3.3 log_10_ reduction in viable counts after 24 h of exposure (Figure 2). All concentrations of SMT19969 resulted in a reduction in viable counts to below the LOD (>3.2 log_10_ reduction in cfu/mL) at 24 h (Figure 3). Killing by SMT19969 was independent of drug concentration.

The viability of *C. difficile* strain 630 when exposed to vancomycin and SMT19969 over 24 h was similar to that observed for *C. difficile* BI1. Vancomycin gradually reduced the viability of *C. difficile* 630 over time in a concentration-independent manner, although a greater reduction in viable counts, compared with *C. difficile* BI1, of 3.0–3.6 log_10_ was observed at 24 h (Table 3). As observed with BI1, SMT19969 was bacteriostatic to 8 h with reductions in viable counts to below the LOD (>3.8 log_10_) at concentrations ≥5 × MIC. Fidaxomicin was more effective at killing *C. difficile* 630 than *C. difficile* BI1, with counts reduced below the LOD by 6 h (20 × MIC) or 24 h (2–10 × MIC).

Against *C. difficile* 5325, vancomycin was bacteriostatic at all concentrations tested (Table 3). Consistent with observations for strains 630 and BI1, all tested concentrations of SMT19969 were bacteriostatic for ∼8 h. Between 8 and 24 h, counts of *C. difficile* 5325 fell by ≥3 log_10_ cfu/mL at concentrations >5 × MIC (Table 3). Fidaxomicin resulted in a marked decrease in viable counts, with concentrations ≥5 × MIC resulting in loss of viability of *C. difficile* to below the LOD by 24 h (Table 3). At 20 × MIC, fidaxomicin rapidly reduced cell viability to below the LOD by 6 h.

### PAE

**Figure 4.**
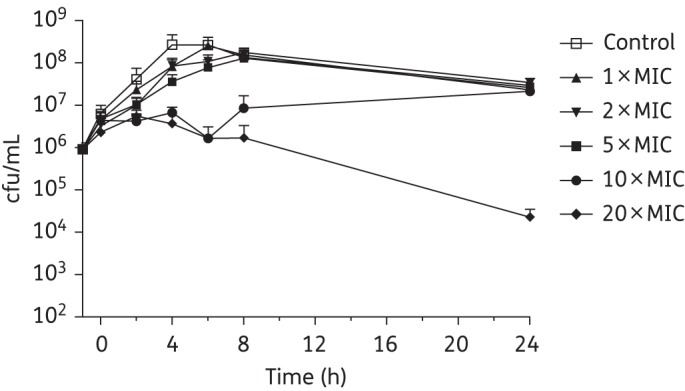
PAE of SMT19969 against *C. difficile* BI1. Data are the means (+SD) of triplicate experiments. Drug removed at 0 h.

The PAE of each antibiotic was established against *C. difficile* BI1, 630 and 5325 by monitoring the growth of each strain following a 1 h exposure to 1, 2, 5, 10 or 20 × MIC of the drug. Recovery of *C. difficile* BI1 following pre-exposure to SMT19969 is shown in Figure 4, with calculated PAEs for all drug–strain combinations shown in Table 4.

**Table 4.** PAE for SMT19969, vancomycin and fidaxomicin against *C. difficile* BI1, 630 and 5325

Strain	Agent	PAE (h)				
		1 × MIC	2 × MIC	5 × MIC	10 × MIC	20 × MIC
BI1	SMT19969	0	0	2	8–20	>20
	vancomycin	0	0	0	0	0
	fidaxomicin	0	8–20	8–20	8–20	NE
5325	SMT19969	0	0	0	4	NE
	vancomycin	0	2	2	0	0
	fidaxomicin	8–20	8–20	8–20	8–20	>20
630	SMT19969	0	2	2	4	>20
	vancomycin	0	0	0	0	2
	fidaxomicin	8–20	8–20	8–20	>20	>20

NE, not established due to inconsistent growth recovery (see the text).

SMT19969 had a PAE of 2 h for *C. difficile* BI1 at 5 × MIC with concentrations of 10 or 20 × MIC suppressing growth for ≥8 h. At 20 × MIC, *C. difficile* BI1 did not recover with a 2 log_10_ reduction in viable counts at 24 h post-removal of SMT19969 (Figure 4). Fidaxomicin had a pronounced PAE (8–20 h) at concentrations ≥2 × MIC. At 20 × MIC of fidaxomicin, inconsistent recovery of bacteria was observed with a 1–2 log_10_ reduction in viability noted in two out of three replicates (complete recovery was seen in the third replicate; Table 4). Vancomycin showed no measurable PAE against *C. difficile* BI1 (Table 4).

At all tested concentrations, a 1 h exposure to vancomycin resulted in a PAE of 0–2 h for *C. difficile* 5325 (Table 4). SMT19969 concentrations of 10 × MIC were required to produce a quantifiable PAE of 4 h. At 20 × MIC of SMT19969, inconsistent recovery of *C. difficile* 5325 was observed (viability in two out of three replicates reduced below the LOD). Fidaxomicin concentrations ≥MIC had a prolonged PAE of 8–20 h. At 20 × MIC, the growth of the culture remained suppressed at 24 h post-removal of antibiotic, with a concomitant 2 log_10_ reduction in viable counts.

For *C. difficile* 630, the PAE of vancomycin was <2 h for concentrations from 1 to 10 × MIC, whilst at 20 × MIC a PAE of 2 h was observed. SMT19969 exhibited a PAE of 2–4 h up to 10 × MIC (Table 4). At 20 × MIC of SMT19969, counts of *C. difficile* 630 fell below the LOD by 24 h post-removal of SMT19969. Fidaxomicin showed a prolonged (8–20 h) PAE against strain 630 at concentrations between 1 and 5 × MIC whilst at higher concentrations no growth was observed with the viability of the culture reduced 2.4 log_10_ in the 24 h following removal of antibiotic.

## Discussion

Current antimicrobial therapy options for CDI are very limited.^19^ Clinical response to metronidazole is inferior at the end of therapy compared with vancomycin in patients with severe CDI.^20,21^ Isolates showing reduced susceptibility to metronidazole have been reported,^16^ and whilst no link between clinical outcome and the MIC of metronidazole has been established, elevated MICs in conjunction with low intraluminal antibiotic concentrations following oral administration may affect efficacy. Both vancomycin and metronidazole are associated with high rates of recurrent disease with 20%–30% of subjects experiencing a recurrent infection following the primary episode; recurrence rates may be >65% following a third episode of CDI.^22^ Recurrent CDI remains frustratingly difficult to treat, with a deleterious impact on patient welfare and healthcare system resources. The recently approved fidaxomicin has been shown in Phase 3 clinical studies to be non-inferior in clinical response to vancomycin at the end of treatment and to be superior in sustained clinical response to 25 days post end of therapy with an overall reduction in rates of recurrence compared with vancomycin. However, recurrence rates were comparable for vancomycin and fidaxomicin for subjects infected with *C. difficile* BI (ribotype 027) strains*.*^23,24^ New agents, particularly those that reduce rates of recurrent disease, are required to effectively manage CDI.

The studies described here show that SMT19969 resulted in potent inhibition of a range of *C. difficile* clinical isolates, consistent with previously reported *in vitro* activity against *C. difficile*.^14,15^ Compared with vancomycin and metronidazole, SMT19969 was considerably more potent; MIC_90_ values were typically 16-fold lower, with consistent activity against a range of clinically relevant ribotypes. Fidaxomicin showed increased potency compared with SMT19969 with MIC_90_ values one dilution and MIC_50_ values two dilutions lower than those recorded for SMT19969.

In addition to potent growth inhibition, SMT19969 was bactericidal against three *C. difficile* clinical isolates representing ribotypes 027, 012 and 078. Against *C. difficile* BI1, SMT19969 resulted in a >3 log_10_ reduction in viable counts following 24 h of exposure, with viable counts typically reduced to below the LOD of the assay; fidaxomicin showed reduced killing at 24 h with typical log_10_ reductions in cfu/mL of 1.5–2.0 (although at 20 × MIC viable counts were reduced by 3.3 log_10_ cfu/mL). Slightly reduced killing by SMT19969 of strain 5325 was observed at concentrations ≤5 × MIC. Against *C. difficile* 630 and 5325, fidaxomicin was bactericidal at all concentrations above the MIC and, consistent with previous reports, killing by fidaxomicin was time dependent.^25^

Killing by vancomycin was largely independent of drug concentration, and in accordance with previous results,^26^ vancomycin was bacteriostatic. In addition, vancomycin was shown to be associated with a minimal PAE (typically 0–2 h), as reported previously.^18,26^ SMT19969, at concentrations ≥5 × MIC, was associated with a pronounced PAE with no growth recovery observed following a 1 h exposure to 20 × MIC. Although higher concentrations of SMT19969 were required to effect a prolonged PAE, mean faecal concentrations of 1363 μg/g (SD ±446), more than three orders of magnitude higher than the MIC_90_ of SMT19969 for *C. difficile*, were observed in Phase 1 healthy volunteers receiving 200 mg twice daily for 10 days.^27^ As previously reported, fidaxomicin showed a very prolonged PAE, typically 8–20 h, at all concentrations.^18^

The data reported here show that SMT19969 has potent and consistent activity against the examined *C. difficile* strains. In addition*,* SMT19969 was bactericidal against all strains tested, and was associated with a pronounced PAE at higher concentrations, as would be expected in the gastrointestinal tract of CDI subjects. In conjunction with its narrow spectrum of activity,^14,15^ these data support continued investigation of SMT19969 as a potential therapy for CDI.

## Funding

This study was initiated and financially supported by Summit plc through a Seeding Drug Discovery Award (grant number 091055) and a Translation Award from the Wellcome Trust (grant number 099444).

## Transparency declarations

S. D. B. has received funding for consultancy research from Procarta Biosystems. M. H. W. has received consulting fees from Actelion, Astellas, Cubist, The Medicines Company, Merck, Novartis, Optimer, Pfizer, Sanofi-Pasteur, Summit, Synthetic Biologics and VH Squared, lecture fees from Alere, Astellas and Pfizer, and grant support from Actelion, Astellas, bioMérieux, Cubist, Da Volterra, Merck and Summit. D. C., A. W., S. B. and P. W. are employees of Evotec and R. J. V. is an employee of Summit plc and holds share options. All other authors: none to declare.
